# (3*R*,3a*S*,6*R*,6a*R*)-3-(1-Nitro­eth­yl)perhydro­furo[3,2-*b*]furan-3,6-diol

**DOI:** 10.1107/S1600536810022774

**Published:** 2010-06-18

**Authors:** Jing-Yu Zhang, Jing Yang

**Affiliations:** a School of Pharmacy Henan University of Traditional Chinese Medicine, Zhengzhou 450008, People’s Republic of China

## Abstract

The mol­ecule of the title compound, C_8_H_13_NO_6_, a sucrose derivative, consists of two fused tetra­hydro­furan rings having the *cis* arrangement at the ring junctions, giving a V-shaped mol­ecule. An intra­molecular O—H⋯O inter­action occurs. Inter­molecular O—H⋯O hydrogen bonds help to stabilize the crystal structure.

## Related literature

For applications of sucrose and its derivatives, see: Chang *et al.* (2001 ▶); Liu *et al.* (2004 ▶); Stutz *et al.* (1999 ▶).
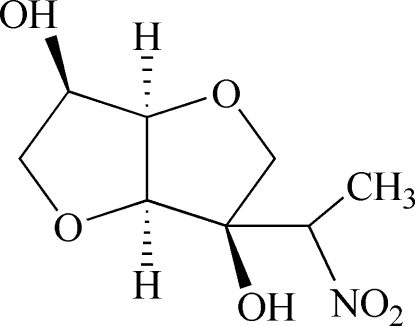


## Experimental

### 

#### Crystal data

C_8_H_13_NO_6_
*M* *_r_* = 219.19
Monoclinic, 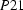

*a* = 6.959 (4) Å
*b* = 5.525 (3) Å
*c* = 12.384 (6) Å
β = 97.077 (7)°
*V* = 472.5 (4) Å^3^
*Z* = 2
Mo *K*α radiation
μ = 0.13 mm^−1^
*T* = 298 K
0.42 × 0.23 × 0.14 mm

#### Data collection

Siemens SMART CCD area-detector diffractometer
Absorption correction: multi-scan (*SADABS*; Sheldrick, 1996 ▶) *T*_min_ = 0.946, *T*_max_ = 0.982
2416 measured reflections
935 independent reflections
743 reflections with *I* > 2σ(*I*)
*R*_int_ = 0.059

#### Refinement

*R*[*F* ^2^ > 2σ(*F* ^2^)] = 0.049
*wR*(*F* ^2^) = 0.129
*S* = 0.98
932 reflections
137 parameters
1 restraint
H-atom parameters constrained
Δρ_max_ = 0.21 e Å^−3^
Δρ_min_ = −0.19 e Å^−3^

### 

Data collection: *SMART* (Siemens, 1996 ▶); cell refinement: *SAINT* (Siemens, 1996 ▶); data reduction: *SAINT*; program(s) used to solve structure: *SHELXS97* (Sheldrick, 2008 ▶); program(s) used to refine structure: *SHELXL97* (Sheldrick, 2008 ▶); molecular graphics: *SHELXTL* (Sheldrick, 2008 ▶); software used to prepare material for publication: *SHELXTL*.

## Supplementary Material

Crystal structure: contains datablocks I, global. DOI: 10.1107/S1600536810022774/jh2141sup1.cif

Structure factors: contains datablocks I. DOI: 10.1107/S1600536810022774/jh2141Isup2.hkl

Additional supplementary materials:  crystallographic information; 3D view; checkCIF report

## Figures and Tables

**Table 1:** Hydrogen-bond geometry (Å, °)

*D*—H⋯*A*	*D*—H	H⋯*A*	*D*⋯*A*	*D*—H⋯*A*
O3—H3⋯O1^i^	0.82	2.06	2.785 (5)	147
O4—H4⋯O3^ii^	0.82	2.05	2.777 (4)	147
O4—H4⋯O1	0.82	2.23	2.655 (4)	113

Symmetry codes: (i) 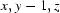; (ii) 
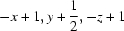
.

**Fig. 1.**
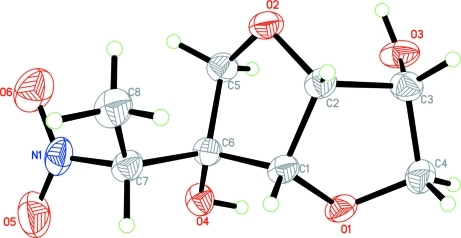
The molecular structure of the compound, with atom labels and 50% probability displacement ellipsoids.

**Fig. 2.**
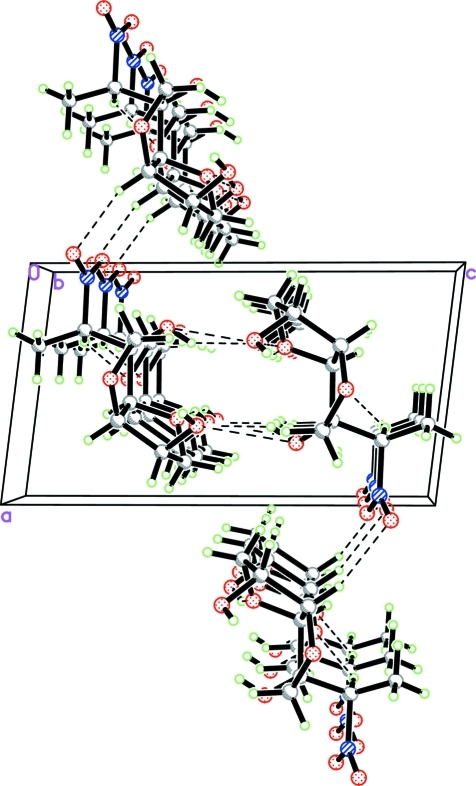
Crystal packing of the title compound, showing a three-dimensional structure, linked by hydrogen bonds(dashed lines).

## supplementary crystallographic information

### Comment

Sugar derivatives are an important class of compounds having a broad spectrum of applications in the chemical, biochemical, medicinal (Chang *et al.*, 2001), and pharmaceutical fields,(Liu *et al.*, 2004; Stutz *et al.*, 1999) Here we report a structure of a novel Sugar derivatives. To develop new applications for sucrose and its derivatives, structural modifications of sucrose have been extensively investigated. As a contribution to the sucrose chemistry, we report here the crystal structure of the title compound.

Molecular structure of title compound is shown in Fig.1. Torsion angle C(6)—C(1)—C(2)—C(3) is -120.4. Intermolecular hydrogen bonds links molecules in crystal structure into three-dimensional structure.

### Experimental

Nitroethane and a catalytic amount of Et3N were added to a stirring solution of 1,4:3,6-dianhydrofructose in EtOH. The mixture was stirred at room temperature for 4 h, and evaporated under reduced pressure to dryness. The residue was recrystallized with EtOH to give title compound as a white crystal.

### Refinement

All H atoms were placed geometrically and treated as riding on their parent atoms with C—H = 0.96 Å (methylene) or 0.93 Å (aromatic), 0.82 Å (hydroxyl) and *U*_iso_(H) =1.2*U*_eq_(C). Because the absolute configuration  was established by the structure determination of a compound containing a chiral reference molecule of known absolute configuration, we have merged the Friedels in the refinement.

### Crystal data

**Table:**

C_8_H_13_NO_6_	*F*(000) = 232
*M**_r_* = 219.19	*D*_x_ = 1.541 Mg m^−^^3^
Monoclinic, *P*21	Mo *K*α radiation, λ = 0.71073 Å
*a* = 6.959 (4) Å	Cell parameters from 895 reflections
*b* = 5.525 (3) Å	θ = 3.0–22.5°
*c* = 12.384 (6) Å	µ = 0.13 mm^−^^1^
β = 97.077 (7)°	*T* = 298 K
*V* = 472.5 (4) Å^3^	Colorless, needlelike
*Z* = 2	0.42 × 0.23 × 0.14 mm

### Data collection

**Table:**

Siemens SMART CCD area-detector diffractometer	935 independent reflections
Radiation source: fine-focus sealed tube	743 reflections with *I* > 2σ(*I*)
graphite	*R*_int_ = 0.059
phi and ω scans	θ_max_ = 25.0°, θ_min_ = 1.7°
Absorption correction: multi-scan (*SADABS*; Sheldrick, 1996)	*h* = −4→8
*T*_min_ = 0.946, *T*_max_ = 0.982	*k* = −6→6
2416 measured reflections	*l* = −14→14

### Refinement

**Table:**

Refinement on *F*^2^	Primary atom site location: structure-invariant direct methods
Least-squares matrix: full	Secondary atom site location: difference Fourier map
*R*[*F*^2^ > 2σ(*F*^2^)] = 0.049	Hydrogen site location: inferred from neighbouring sites
*wR*(*F*^2^) = 0.129	H-atom parameters constrained
*S* = 0.98	*w* = 1/[σ^2^(*F*_o_^2^) + (0.0843*P*)^2^] where *P* = (*F*_o_^2^ + 2*F*_c_^2^)/3
932 reflections	(Δ/σ)_max_ < 0.001
137 parameters	Δρ_max_ = 0.21 e Å^−^^3^
1 restraint	Δρ_min_ = −0.19 e Å^−^^3^

### Special details

**Table:**

Geometry. All e.s.d.'s (except the e.s.d. in the dihedral angle between two l.s. planes) are estimated using the full covariance matrix. The cell e.s.d.'s are taken into account individually in the estimation of e.s.d.'s in distances, angles and torsion angles; correlations between e.s.d.'s in cell parameters are only used when they are defined by crystal symmetry. An approximate (isotropic) treatment of cell e.s.d.'s is used for estimating e.s.d.'s involving l.s. planes.
Refinement. Refinement of *F*^2^ against ALL reflections. The weighted *R*-factor *wR* and goodness of fit *S* are based on *F*^2^, conventional *R*-factors *R* are based on *F*, with *F* set to zero for negative *F*^2^. The threshold expression of *F*^2^ > σ(*F*^2^) is used only for calculating *R*-factors(gt) *etc*. and is not relevant to the choice of reflections for refinement. *R*-factors based on *F*^2^ are statistically about twice as large as those based on *F*, and *R*- factors based on ALL data will be even larger.

### Fractional atomic coordinates and isotropic or equivalent isotropic displacement parameters (Å^2^)

**Table:**

	*x*	*y*	*z*	*U*_iso_*/*U*_eq_	
N1	0.9397 (6)	0.4876 (9)	0.8645 (3)	0.0565 (11)	
O1	0.3548 (4)	0.4642 (5)	0.61605 (19)	0.0468 (7)	
O2	0.5198 (4)	−0.0131 (5)	0.7607 (2)	0.0485 (7)	
O3	0.3353 (5)	−0.0495 (6)	0.55654 (18)	0.0586 (9)	
H3	0.3836	−0.1713	0.5865	0.088*	
O4	0.7338 (4)	0.5239 (6)	0.6667 (2)	0.0475 (8)	
H4	0.6696	0.5192	0.6066	0.071*	
O5	1.0171 (6)	0.6770 (9)	0.8438 (3)	0.0869 (13)	
O6	1.0297 (6)	0.3057 (9)	0.8937 (3)	0.0867 (12)	
C1	0.4230 (5)	0.3930 (7)	0.7261 (3)	0.0402 (10)	
H1	0.3753	0.5017	0.7795	0.048*	
C2	0.3545 (6)	0.1339 (7)	0.7386 (3)	0.0419 (9)	
H2	0.2752	0.1239	0.7986	0.050*	
C3	0.2290 (7)	0.0763 (8)	0.6291 (3)	0.0469 (10)	
H3A	0.1108	−0.0111	0.6407	0.056*	
C4	0.1837 (6)	0.3261 (9)	0.5835 (3)	0.0512 (11)	
H4A	0.1558	0.3209	0.5049	0.061*	
H4B	0.0732	0.3949	0.6133	0.061*	
C5	0.6784 (6)	0.1117 (8)	0.7223 (4)	0.0463 (10)	
H5A	0.6822	0.0785	0.6456	0.056*	
H5B	0.8001	0.0608	0.7625	0.056*	
C6	0.6445 (5)	0.3804 (7)	0.7401 (3)	0.0390 (9)	
C7	0.7219 (5)	0.4776 (9)	0.8547 (3)	0.0454 (10)	
H7	0.6749	0.6440	0.8596	0.055*	
C8	0.6571 (6)	0.3377 (10)	0.9479 (3)	0.0553 (12)	
H8A	0.7002	0.1730	0.9450	0.083*	
H8B	0.5184	0.3413	0.9429	0.083*	
H8C	0.7115	0.4094	1.0154	0.083*	

### Atomic displacement parameters (Å^2^)

**Table:**

	*U*^11^	*U*^22^	*U*^33^	*U*^12^	*U*^13^	*U*^23^
N1	0.056 (2)	0.072 (3)	0.0384 (18)	0.008 (2)	−0.0049 (16)	−0.008 (2)
O1	0.0563 (16)	0.0408 (16)	0.0409 (14)	0.0072 (14)	−0.0031 (12)	0.0095 (13)
O2	0.0592 (17)	0.0359 (16)	0.0496 (15)	0.0079 (14)	0.0034 (13)	0.0026 (14)
O3	0.099 (2)	0.0404 (17)	0.0355 (14)	0.0131 (17)	0.0040 (15)	0.0019 (14)
O4	0.0544 (16)	0.0548 (19)	0.0330 (13)	−0.0059 (15)	0.0037 (12)	−0.0004 (13)
O5	0.072 (2)	0.102 (3)	0.081 (3)	−0.026 (3)	−0.012 (2)	0.000 (2)
O6	0.069 (2)	0.099 (3)	0.090 (3)	0.029 (2)	0.001 (2)	−0.007 (3)
C1	0.051 (2)	0.039 (2)	0.0301 (18)	0.010 (2)	0.0039 (16)	0.0002 (16)
C2	0.047 (2)	0.043 (2)	0.0353 (19)	0.004 (2)	0.0038 (16)	0.0008 (18)
C3	0.057 (2)	0.042 (2)	0.041 (2)	−0.003 (2)	0.0034 (18)	0.0019 (18)
C4	0.052 (3)	0.054 (3)	0.044 (2)	0.003 (2)	−0.0067 (18)	−0.001 (2)
C5	0.051 (2)	0.042 (2)	0.046 (2)	0.007 (2)	0.0061 (18)	−0.0064 (19)
C6	0.050 (2)	0.038 (2)	0.0289 (19)	−0.0013 (18)	0.0054 (16)	−0.0012 (15)
C7	0.052 (2)	0.048 (2)	0.0360 (18)	0.008 (2)	0.0031 (16)	−0.002 (2)
C8	0.070 (3)	0.064 (3)	0.033 (2)	0.006 (3)	0.0090 (18)	0.0003 (19)

### Geometric parameters (Å, °)

**Table:**

N1—O6	1.215 (6)	C2—H2	0.9800
N1—O5	1.219 (6)	C3—C4	1.509 (6)
N1—C7	1.506 (5)	C3—H3A	0.9800
O1—C4	1.430 (5)	C4—H4A	0.9700
O1—C1	1.442 (4)	C4—H4B	0.9700
O2—C2	1.408 (5)	C5—C6	1.523 (6)
O2—C5	1.431 (5)	C5—H5A	0.9700
O3—C3	1.415 (5)	C5—H5B	0.9700
O3—H3	0.8200	C6—C7	1.550 (5)
O4—C6	1.407 (5)	C7—C8	1.504 (6)
O4—H4	0.8200	C7—H7	0.9800
C1—C2	1.523 (6)	C8—H8A	0.9600
C1—C6	1.531 (5)	C8—H8B	0.9600
C1—H1	0.9800	C8—H8C	0.9600
C2—C3	1.552 (5)		
			
O6—N1—O5	123.2 (4)	O1—C4—H4B	110.8
O6—N1—C7	118.1 (5)	C3—C4—H4B	110.8
O5—N1—C7	118.7 (4)	H4A—C4—H4B	108.9
C4—O1—C1	106.6 (3)	O2—C5—C6	106.4 (3)
C2—O2—C5	107.6 (3)	O2—C5—H5A	110.5
C3—O3—H3	109.5	C6—C5—H5A	110.5
C6—O4—H4	109.5	O2—C5—H5B	110.5
O1—C1—C2	106.4 (3)	C6—C5—H5B	110.5
O1—C1—C6	109.2 (3)	H5A—C5—H5B	108.6
C2—C1—C6	105.6 (3)	O4—C6—C5	111.5 (3)
O1—C1—H1	111.8	O4—C6—C1	114.8 (3)
C2—C1—H1	111.8	C5—C6—C1	101.5 (3)
C6—C1—H1	111.8	O4—C6—C7	105.3 (3)
O2—C2—C1	107.7 (3)	C5—C6—C7	115.3 (3)
O2—C2—C3	114.2 (3)	C1—C6—C7	108.6 (3)
C1—C2—C3	104.7 (3)	C8—C7—N1	110.5 (3)
O2—C2—H2	110.0	C8—C7—C6	114.9 (4)
C1—C2—H2	110.0	N1—C7—C6	108.6 (3)
C3—C2—H2	110.0	C8—C7—H7	107.5
O3—C3—C4	108.2 (3)	N1—C7—H7	107.5
O3—C3—C2	111.9 (3)	C6—C7—H7	107.5
C4—C3—C2	102.0 (3)	C7—C8—H8A	109.5
O3—C3—H3A	111.4	C7—C8—H8B	109.5
C4—C3—H3A	111.4	H8A—C8—H8B	109.5
C2—C3—H3A	111.4	C7—C8—H8C	109.5
O1—C4—C3	104.7 (3)	H8A—C8—H8C	109.5
O1—C4—H4A	110.8	H8B—C8—H8C	109.5
C3—C4—H4A	110.8		
			
C4—O1—C1—C2	27.7 (4)	O2—C5—C6—C7	85.8 (4)
C4—O1—C1—C6	141.2 (3)	O1—C1—C6—O4	24.1 (4)
C5—O2—C2—C1	−22.0 (4)	C2—C1—C6—O4	138.1 (3)
C5—O2—C2—C3	93.8 (4)	O1—C1—C6—C5	−96.3 (3)
O1—C1—C2—O2	117.5 (3)	C2—C1—C6—C5	17.7 (3)
C6—C1—C2—O2	1.5 (4)	O1—C1—C6—C7	141.7 (3)
O1—C1—C2—C3	−4.4 (4)	C2—C1—C6—C7	−104.3 (4)
C6—C1—C2—C3	−120.4 (3)	O6—N1—C7—C8	40.8 (5)
O2—C2—C3—O3	−20.6 (5)	O5—N1—C7—C8	−138.7 (4)
C1—C2—C3—O3	96.9 (4)	O6—N1—C7—C6	−86.2 (4)
O2—C2—C3—C4	−136.1 (4)	O5—N1—C7—C6	94.4 (5)
C1—C2—C3—C4	−18.6 (4)	O4—C6—C7—C8	−175.5 (3)
C1—O1—C4—C3	−40.5 (4)	C5—C6—C7—C8	−52.1 (5)
O3—C3—C4—O1	−82.4 (4)	C1—C6—C7—C8	61.0 (5)
C2—C3—C4—O1	35.8 (4)	O4—C6—C7—N1	−51.2 (4)
C2—O2—C5—C6	34.2 (4)	C5—C6—C7—N1	72.2 (4)
O2—C5—C6—O4	−154.1 (3)	C1—C6—C7—N1	−174.7 (3)
O2—C5—C6—C1	−31.3 (4)		

### Hydrogen-bond geometry (Å, °)

**Table:**

*D*—H···*A*	*D*—H	H···*A*	*D*···*A*	*D*—H···*A*
O3—H3···O1^i^	0.82	2.06	2.785 (5)	147
O4—H4···O3^ii^	0.82	2.05	2.777 (4)	147
O4—H4···O1	0.82	2.23	2.655 (4)	113

Symmetry codes: (i) *x*, *y*−1, *z*; (ii) −*x*+1, *y*+1/2, −*z*+1.
